# Risk factors and prognosis of visual and cranial nerve deficits in patients operated for pituitary tumor – with a focus on intrasellar pressure

**DOI:** 10.1007/s00701-025-06668-4

**Published:** 2025-10-06

**Authors:** Gabriel Simander, Peter Lindvall, Per Dahlqvist, Lars-Owe D. Koskinen

**Affiliations:** 1 Department of Clinical Sciences - Neurosciences, Umeå University, Umeå, Sweden; 2 Department of Public Health and Clinical Medicine, Section of Endocrinology and Diabetes, Umeå University, Umeå, Sweden

**Keywords:** Intrasellar pressure, Pituitary tumor, Visual, Cranial nerve

## Abstract

**Purpose:**

The aim of this study was to evaluate if intrasellar pressure (ISP) is associated with the risk of visual impairment in patients with a pituitary tumor, and the prognosis for visual function after tumor surgery.

**Method:**

Retrospective, single-center study including 100 consecutive patients operated for a pituitary tumor, who had their ISP measured. Data on patient and tumor characteristics, pre- and postoperative visual acuity, visual fields, and cranial nerve III, IV, and VI deficits were collected from patient files.

**Results:**

Before surgery, 64% had visual acuity impairment and 65% visual field deficits. Postoperatively, the frequencies were 40% for visual acuity impairment and 41% for visual field deficits. Risk factors for preoperative visual impairment were tumor volume, suprasellar tumor extension (SIPAP grade 3–4), and female sex. ISP was associated with higher risk of visual acuity impairment at postoperative follow up. No other correlations between ISP and pre- or postoperative visual and cranial nerve outcome were found. Age was associated with lower chance of visual acuity improvement and increased risk of visual field deficits postoperatively.

**Conclusion:**

Overall, ISP does not seem to play an important role as a risk factor or prognostic factor for visual and cranial nerve impairment in pituitary tumor disease. However, ISP showed an association with postoperative visual acuity impairment. The clinical relevance of this results is not straight-forward. Tumor size, suprasellar growth pattern, and female sex are confirmed risk factors for preoperative visual symptoms. High age appears to negatively influence visual outcome after surgery.

## Background

Pituitary tumors are among the most common central nervous system neoplasms, with most being benign. Their clinical incidence is approximately 4–7 per 100,000 annually[9, 33, 37]. Located in the confined space of the sella turcica, pituitary tumors often cause symptoms by compressing adjacent structures, such as the optic apparatus or cranial nerves (CNs) III, IV, V, and VI[1, 16, 33, 36].

Common symptoms include visual deficits, headaches, and hormonal imbalances[36, 37]. In non-functioning pituitary adenomas (NFPAs), 60–80% of patients with macroadenomas (> 10 mm) present with visual deficits[10]. Impairment of visual acuity and visual fields may arise from tumor pressure on the optic chiasm, which lies above the sella[11], and lateral extension into the cavernous sinus may also affect eye movement and pupil function via CN involvement[29]. Surgery is the primary treatment for symptomatic NFPAs, with visual symptoms being the most common indication[5, 6, 13, 22].

Few studies have investigated intrasellar pressure (ISP) in pituitary tumor patients, in which ISP has been linked with tumor size, headache, hormonal dysfunction, and quality of life[2, 18, 19, 41, 42]. While two studies examined ISP in relation to visual symptoms, they neither focused on this as a primary outcome nor found any clear association[19, 37].

There is thus a gap in science that we have sought to address**.** This study aims to determine whether ISP correlates with pre- or postoperative visual function—specifically visual acuity, visual fields, and CN III, IV, and VI involvement. We hypothesized that high ISP may increase the risk of preoperative deficits and predict worse outcome. Secondary objectives include analyzing visual symptoms in relation to tumor and patient characteristics.

## Method

### Study population

This study was a single-center, retrospective, observational analysis based on a consecutively collected cohort, with ISP data obtained prospectively. We included patients who underwent surgery for suspected pituitary adenoma at the Department of Neurosurgery, Umeå University Hospital, Umeå, Sweden, between 2009 and 2015. Intraoperative ISP measurements were performed as routine during these years, resulting in a final cohort of 100 consecutively included patients. All surgeries were performed by the same neurosurgeon as responsible.

### Exclusions

#### Unreliable ISP measurement

In one patient, the sellar walls were too damaged for accurate measurement of ISP; this patient was hence excluded from all analyses.

#### Preexisting eye conditions

For the analysis of ISP in relation to visual acuity, patients with pre-existing ocular conditions documented in their medical records were excluded to ensure that any observed visual impairments were attributable to the pituitary tumor rather than other causes. For the analysis of ISP in relation to changes in visual acuity after pituitary surgery, the patients excluded due to preexisting eye conditions were re-included, as a postoperative improvement is likely to be a direct result of the surgery and therefore related to the pituitary tumor. In analysis of visual fields, a few patients, with preexisting eye conditions that made conclusive examination impossible, were initially excluded.

**Fig. 1:**
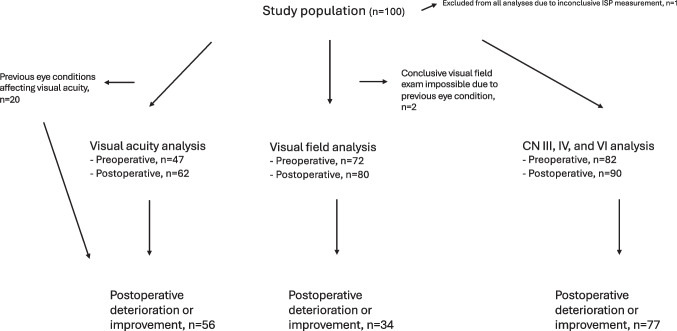
Flowchart illustrating study populations and exclusions

Figure 1 illustrates the study population in the different analyses after exclusions and missing data are accounted for.

### Data collection

A standard clinical work-up was applied for all patients. This included a neuroophthalmological examination conducted by an ophthalmologist, a neuroendocrinology evaluation by an endocrinologist, neuroradiology examination with MRI (magnetic resonance imaging) evaluated by a neuroradiologist, and a clinical neurological examination by a neurosurgeon, prior to surgery.

After surgery, the patients were re-examined at a 3-month postoperative follow-up, undergoing the same protocol of examinations as before surgery. Since this constituted a routine clinical standard, clinical information was not blinded, but ISP was not known by others than the neurosurgeon. Data necessary to conduct the analyses were collected from patients’ electronic files, pre- and postoperatively. This included basic patient characteristics including age and sex, previous diseases and previous eye-related conditions, ISP, neuroophthalmological parameters, laboratory parameters, immunohistopathological diagnose and radiological images.

In case of paradoxical results or difficulties with compliance at the examination as reported by the ophthalmologist, data were considered as inconclusive and thus defined as missing. Naturally, in comparisons of preoperative and postoperative results, only patients for whom the results from both examinations were available were included in the analysis, otherwise classified as missing. In case of unavailable data, this was also defined as missing.

### Tumor characteristics

Tumors were classified anatomically from the preoperative MRI, using the SIPAP- and Knosp-classifications[12, 25]. Tumor volumes were calculated from preoperative MRI using Automatic Sectra Volume Tool (Sectra Workstation, IDS7, v. 23.1). The tumors were classified biochemically by reviewing the opinion from the endocrinologist, the clinically laboratory results together with after the report from immunohistopathologic analysis of the tumor.

### Neuroophthalmological parameters

#### Visual acuity

Visual acuity was measured using a Snellen chart and expressed on a decimal scale, ranging from 0.1–2.0, with ≥ 1.0 considered normal. It was measured using patients’ own correction (glasses), if applicable. The change in visual acuity of each patient (preoperatively vs. postoperatively) was classified as improvement, deterioration, or no change. A change of at least 0.1 for at least one eye was required to be defined as improvement or deterioration. Complete recovery of visual acuity was defined as 1.0 or more.

#### Visual fields

The vast majority of examinations were made using the Goldmann perimeter and the Humphrey visual field analyzer, with a few made using frequency doubling technology perimetry. The visual field examinations were sorted into four different categories (0–III) based on the number of quadrants, uni- or bilateral deficits, and the interpretation of the ophthalmologist reviewing the examination.

0 = No deficits.

I = 1 quadrant deficit.

II =  > 1 quadrant unilateral deficit.

III =  > 1 quadrant bilateral deficit.

Change in visual field deficits (preoperatively vs. postoperatively) was defined by the opinion of the ophthalmologist, and classified as amelioration, deterioration, or no change.

#### Cranial nerves III, IV, and VI

The functions of CNs III, IV, and VI were evaluated in the standard neurological examination performed by a neurosurgeon preoperatively, but also in the neuroophthalmological examination performed pre- and postoperatively. Aberration from normal was considered an impact on the examined nerve. Regarding the impact on CNs III, IV, and VI, all patients who had nerve impact according to medical records were considered to have confirmed nerve impact. For all other patients, we concluded that there was no nerve impact, whether or not this was explicitly stated.

#### ISP measurements

ISP measurements were included as part of the standard surgical routine at the clinic and a standard clinical work-up was applied for all patients. The surgical approach was transsphenoidal tumor extirpation, either microscope assisted through lateral rhinotomy, or by endoscopic assisted transnasal approach. Surgery was performed under standard neuro-anesthesia with oral intubation. Patients were normoventilated with pCO2-levels between 4.6–5.5 kPa. During surgery, before the tumor resection was initiated, a small bone window, maximum 2 mm, was made in the floor of the sella, followed by a 1–2 mm sharp cut in the dura. A Codman® Microsensor™ was then inserted into the intrasellar room to measure the ISP. The opening was made just large enough for insertion of the sensor, to avoid leakage of the intrasellar content. The ISP values were determined after pressure fluctuations had settled, in the majority of cases approximately 30 s after insertion, and documented in the operative report. The accuracy of the measuring device is well documented, and it is used clinically as a standard method for intracranial pressure monitoring[26–28].

### Definition of risk factors

We chose to include the following potential risk factors in the analysis: ISP, tumor volume, age, and sex. For visual acuity and visual fields, we also included suprasellar tumor extension defined as SIPAP grade 3–4. As regards CNs III, IV, and VI deficits, we instead included parasellar invasiveness defined as Knosp III–IV. For analyses of postoperative visual acuity and visual field impairment, we also included preoperative impairment as a potential risk factor.

### Statistical analysis

Data are presented as mean ± standard deviation and median (interquartile range; IQR). For group comparisons, independent two-sided t-tests were performed for continuous variables, and chi-squared tests for nominal variables. For testing difference of mean ISP between more than two groups Oneway Analysis of Variance (ANOVA) was used. The chosen potential risk factors (ISP, age, sex, tumor volume, tumor invasiveness and when applicable preoperative deficits) were analyzed in a multivariable logistic regression analysis, with results presented as odds ratios (OR) and unit odds ratios (uOR). The potential risk factors included in the multivariable regression model were chosen on clinical basis. Because several of the risk factors are known to potentially correlate to each other they were primarily included in the multivariable model. No correction for multiple testing was performed. Cases of non-conclusive examinations or non-available data were registered as “missing” in the analysis. P < 0.05 was considered statistically significant. JMP 17.0 (SAS Institute Inc., Cary, NC, USA) was used for statistical analyses.

### Ethics

The study was approved by the Regional Ethics Review Board. It was conducted in accordance with the World Medical Association’s Declaration of Helsinki, Ethical Principles for Medical Research Involving Human Subjects. The surgical procedure that all subjects have undergone was the routine surgical treatment option at the clinic, before which conventional informed verbal consent was obtained. For this retrospective study, including data collection and analysis, no further consent was needed. This was approved by the Ethics Review Board.

## Results

### Patient and tumor characteristics

Basic characteristics of the patients and tumors are presented in Table 1. The study population after initial exclusions consisted of 99 patients (54 male and 45 female) with a mean age of 60 yrs. The mean ISP was 23.5 ± 8.9 (median 22, IQR 17–28) mmHg. There were no correlations found when analyzing ISP, tumor volume, and tumor invasiveness, respectively, in relation to age and sex. The biochemical diagnoses of the study population are presented in Table 2*.*

**Table 1:** Patient and tumor characteristics

	n	Mean Age		Mean ISP		Tumor volume		Parasellar invasive		S grade 3–4	
		(yrs)		(mmHg)		(cm^3)		(Knosp III-IV)		(SIPAP)	
Total	99	60.4 ± 14.8		23.5 ± 8.9		6.1 ± 5.4		38 (38%)		68 (69%)	
			*t-test*		*t-test*		*t-test*		*Chi2*		*Chi2*
Men	54	61.7 ± 14.0		25.1 ± 9.1		6.4 ± 5.8		22 (42%)		35 (65%)	
Women	45	58.9 ± 15.8	*p* = *0.35*	21.6 ± 8.4	p = 0.052	5.8 ± 4.9	*p* = *0.61*	16 (37%)	*p* = *0.67*	33 (75%)	*p* = *0.27*

**Table 2:** Biochemical diagnoses in the study population after initial exclusion

Biochemical diagnosis	n
Acromegaly	9
Gonadotrophin-producing	1
Prolactinoma	4
ACTH-producing	0
TSH-producing	0
NFPA	79
Non-adenoma	6
Total	99

Table 1 shows the basic characteristics of the included patients and tumors. T-tests (continuous variables) and chi-squared tests (nominal variables) were used to analyze any differences between males and females.

### Excluded patients

Twenty patients had a history of co-existing eye conditions that were considered to potentially affect visual acuity. These patients were therefore excluded from analyses of preoperative- and postoperative visual acuity. The eye-related comorbidities are listed in Table 3. Two patients were excluded from visual field analysis due to preexisting conditions, one with visual field disturbances since childhood and one with to visual field disturbances after repeated eye surgery. As mentioned above, 1 patient was primarily excluded from all analyses due to inconclusive ISP-measurement, *see *Fig. 1*.*

**Table 3:** Co-existing eye conditions

**Condition**	n
Cataract surgery	4
Cataract	3
Suspected glaucoma	3
Severely impaired vision/blind	3
Vitreous detachment	2
Maculopathy/retinopathy	1
Corneal ulcer	1
Dry AMD	1
Laser-treated PCO	1
Epiretinal membrane	1
Eye migraine	1
Diphtheria	1
Conjunctival cyst surgery	1

### Missing data

The numbers of unavailable or inconclusive data, thus reported as missing were as follows for the separate analyses: Preoperative visual acuity, n = 32; postoperative visual acuity, n = 17; change in visual acuity (reliable both pre- and postoperative data needed), n = 43; preoperative visual fields, n = 25; postoperative visual fields, n = 17; change in visual fields (reliable both pre- and postoperative data needed), n = 63; preoperative CN III, IV, VI -deficit, n = 17; postoperative CN III, IV, VI -deficit, n = 9; change in CN III, IV, VI-deficit, n = 22. Figure 1 illustrates the sizes of the subgroups for each analysis, after accounting for excluded cases and missing data.

### Neuroophthalmological outcome

The respective impacts of the analyzed risk factors on visual acuity, visual field impairment, and CN deficits are presented in Table 4.

**Table 4:** Risk factors for visual and cranial nerve impairment and improvement

**Visual symptoms**	n	*Age (yrs)*		*Sex (female)*		*ISP (mmHg)*		*Tumor volume (cm*^*3*^*)*		*S (SIPAP) 3–4*		*Preoperative impairment*	
		OR	p	OR	p	OR	p	OR	p	OR	p	OR	p
												Visual acuity	
Preoperative visual acuity deficit	30/47 (64%)	1.05	0.19	**7.27**	** < 0.0001**	0.89	0.074	**2.18**	**0.0004**	**6.44**	**0.0007**		
Postoperative visual acuity deficit	25/62 (40%)	1.03	0.538	2.39	0.517	**1.20**	**0.031**	**1.41**	**0.023**	0.50	0.641	**30.7**	**0.005**
Visual acuity improvement	23/36 (64%)	**0.93**	**0.033**	2.71	0.140	0.99	0.95	1.10	0.22	2.48	0.49		
Preoperative visual field deficit	47/72 (65%)	0.99	0.98	**5.56**	**0.007**	1.03	0.41	**1.19**	**0.041**	**4.36**	**0.020**		
Preoperative bilateral deficit (grade III)	35/72 (48%)	1.002	0.89	**5.02**	**0.005**	1.07	0.061	1.07	0.22	**4.28**	**0.027**		
												Visual field	
Postoperative visual field deficit	33/80 (41%)	**1.10**	**0.0015**	3.14	0.12	1.02	0.061	1.08	0.26	0.33	0.20	**16.1**	**0.0006**
Visual field improvement	27/34 (79%)	0.98	0.64	0.49	0.48	0.93	0.16	1.19	0.24	4.18	0.27		
										Knosp III–IV			
										OR	p		
CN III, IV, VI deficit preoperatively	8/82 (10%)	1.004	0.89	4.56	0.0851	0.51	1.02	0.49	1.05	3.10	0.23		
CN III, IV, VI improvement	5/8 (63%)	1.01	0.78	7.1	0.074	1.04	0.56	0.92	0.49	2.6	0.39		

Table 4 multivariable logistic regression analysis showing the impact of the patient and tumor characteristics on pre- and postoperative visual acuity, visual fields, and cranial nerve (CN) III, IV, and VI impairment and improvement after surgery. Significant p-values (< 0.05) and corresponding odds ratios) are marked in bold. OR: odds ratio, uOR = unit odds ratio, CN = cranial nerve.

### Visual acuity

Preoperative visual acuity impairment was found in 30 of 47 (64%) of the analyzed patients. The multivariable regression analysis showed that the risk of preoperative visual acuity impairment increased with tumor volume (unit odds ratio, uOR 2.18, p = 0.0004). The risk was higher in females (OR 7.27, p < 0.0001), and in patients with high grade of cranio-caudal tumor extension, i.e., suprasellar SIPAP grade 3–4 (OR 6.44, p = 0.0007).

Postoperative visual acuity impairment was found in 25 of 62 (40%). The risk of postoperative visual acuity impairment was correlated with ISP (uOR 1.20, p = 0.0308) and tumor volume (uOR 1.41, p = 0.0233), but most strongly with preoperative impairment (OR 30.7, p = 0.0052). Age and sex were not found to be associated with postoperative visual acuity impairment.

After re-inclusion of patients earlier excluded due to eye-related co-morbidity, analysis of change in visual acuity after pituitary surgery showed improvement in 23 of 36 (64%) patients who had some preoperative impairment. Deterioration was found in 11of 56 (20%) patients at the postoperative control. Age was shown as a negative predictive factor for the chance of improvement (uOR 0.93, p = 0.033). No other risk factor including ISP correlated to the chance of improvement. None of the analyzed factors was significantly associated with risk of postoperative deterioration of visual acuity.

### Visual fields

**Fig. 2:**
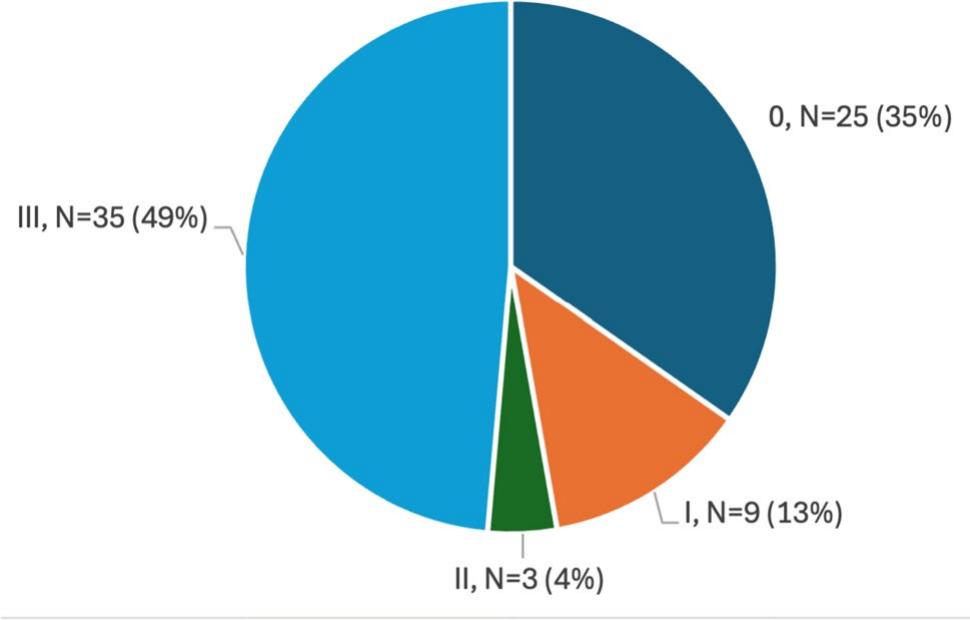
Distribution of visual field deficit categories

Before surgery, 47 of 72 (65%) patients had some sort of visual field deficit. The distribution of grades is presented in Fig. 2. Preoperative visual field deficits were more common in females (78%, 25/32) than in males (55%, 22/40) (p = 0.0493). ISP was not correlated with occurrence of visual field deficit (p = 0.30, t-test), nor was there any difference in ISP between the groups based on severity of deficits (p = 0.37, ANOVA). In the multivariable regression analysis, female sex (OR 5.56, p = 0.0067), large tumor volume (uOR 1.19, p = 0.041), and SIPAP grade 3–4 (OR 4.36, p = 0.020) were associated with increased risk of preoperative visual field deficit. Significant risk factors for grade 3, i.e., bitemporal visual field deficits, were female sex (OR 5.02, p = 0.0053) and SIPAP grade 3–4 (OR 4.28, p = 0.0273).

After surgery, 27 of 34 (79%) of the patients with preoperative visual field deficits had improved and 2 (6%) had deteriorated. Among all 80 patients undergoing a postoperative visual field examination, 33 (41%) had a visual field deficit. A multivariable analysis also including preoperative visual field deficits showed that age (uOR 1.10, p = 0.0015) and a preoperative visual field deficit (OR 16.1, p = 0.0006) were risk factors for having a visual field deficit postoperatively. ISP did not correlate significantly to the risk of postoperative visual fields deficits in the multivariable analysis, nor when evaluated as univariable predictor. None of the analyzed factors showed any significant correlation with visual field improvement or deterioration after surgery.

### CN III, IV, or IV deficits

Preoperatively, 8 of 82 (10%) of the patients had a CN III, IV, or VI deficit. An increased incidence of CN deficits was seen in patients with parasellar-invasive tumors (Knosp III–IV) (p = 0.0425, chi-squared). In the multivariable regression analysis including the defined potential risk factors, none showed any significant effect on the risk of preoperative CN deficits.

After surgery, 5 of the 8 (62%) patients with a preoperative CN function deficit had improved function, and one new CN deficit had occurred. Improved CN function after pituitary surgery was not significantly correlated with age, sex, ISP, tumor volume, or parasellar tumor extension.

## Discussion

This study investigated risk factors for visual and cranial nerve impairment before and after pituitary tumor surgery, with a focus on ISP. We found that elevated ISP showed an association with the risk of postoperative visual acuity loss, but not with preoperative visual acuity, preoperative visual field deficits, or postoperative visual fields. ISP was also not linked to the likelihood of visual improvement after surgery. Overall, this suggests that ISP is not a major predictor of visual symptoms or outcomes following decompressive surgery.

Suprasellar tumor extension and larger tumor volume were confirmed as risk factors for reduced visual acuity and visual field deficits preoperatively. Female sex was associated with preoperative visual impairment but did not influence postoperative outcomes. Age was linked to an increased risk of postoperative visual field deficits and lower chance of improvement of visual acuity after surgery. In univariate analysis, parasellar growth correlated with higher risk of cranial nerve III, IV, or VI deficits, but this was not supported in multivariable analysis. In this cohort, 64% of the patients had impaired visual acuity, 65% had visual field deficits, and 10% had cranial nerve involvement. These findings are consistent with previous reports, despite variability in the literature. A review of 1,307 cases found visual field deficits in 31–86% and cranial nerve III palsies in up to 15% of patients[14, 23, 37].

Adenoma size and high levels of cranio-caudal growth are well-established risk factors for visual symptoms in pituitary tumor disease [4, 20, 30, 31, 39]. Age has previously been reported as a risk factor for worse visual outcomes, associations partially confirmed in this cohort [20, 30, 31, 39]. The underlying reason for female sex as a risk factor for visual impairment remains unclear. Given the small sample size, there is a risk of overestimating this association. However, prior studies have reported higher rates of visual deficits and blindness in females. In contrast, males are often diagnosed later, typically with larger tumor volumes. Some studies have also shown worse surgical outcomes in females with NFPA [3, 32, 38]. While differences in tumor aggressiveness have been proposed as a possible explanation, this remains unsubstantiated [3, 38]. Notably, our study found no sex-based differences in tumor volume or suprasellar extension.

There are no previous studies comparing ISP to the level of visual acuity impairment or the impact on CN III, IV, or VI. Regarding ISP in relation to visual field deficits, there are only two previous studies touching on the subject. Periera-Neto et al*.*[37] investigated the correlation between ISP and the extent of the affected visual quadrants in 25 patients and found no significant relationship. Hayashi et al*.*[19] performed intraoperative pressure measurements on 108 patients undergoing endoscopic surgery for pituitary adenoma. They reported that the ISP for patients with visual disturbances (17.8 ± 7.1 mmHg) did not differ significantly from that in patients with no symptoms (14.2 ± 4.7 mmHg). They showed higher ISP for patients with diplopia (32.3 ± 12.3 mmHg) but reported no further analysis of visual symptoms in relation to ISP. Results from our more extended study are in line with the findings of Periera-Neto et al.

Different mechanisms for how pressure may affect nerve cells have been suggested. Some of these are ischemia, oedema, and disorganized cytoskeleton – mechanisms which all carry a potential for relatively quick recovery. Demyelination has also been described secondary to pressure elevation. Recovery from this would require remyelination, which may explain the delayed recovery sometimes seen after decompression[7, 8, 44]. In one of our previous studies, based on the same population, we showed that a parasellar tumor growth pattern was associated with higher ISP[42]. One suggested reason behind this phenomenon may be that a higher pressure is required for a tumor to grow in the parasellar direction against the cavernous sinus and carotid arteries, compared with cranially against the sellar diaphragm and the optic chiasm. This could hypothetically explain why elevated ISP is not shown to be a risk factor involved in visual acuity or visual field impairments.

All studies have merits and weaknesses. This study covers some novel aspects and research questions that have not been addressed in the existing literature. The study population was derived from a single center that serves a large geographical region, and ISP was measured standardized, using a consecutive and prospective approach. These methodological features increase internal validity and minimize the risk of selection bias. Due to the nature of the study, only patients who underwent surgical treatment were included. As a result, smaller, non-progressive, or asymptomatic tumors, and most of the prolactinomas managed medically, were not included. However, the fact that inclusion of patients was based on a variety of surgical indications, rather than solely those with large tumor volumes, contributes to the heterogeneity of the study population. This diversity may partially mitigate the selection bias introduced by limiting the sample to surgically treated cases.

The retrospective nature of this study entails certain limitations. Different perimetric methods were used, each with recognized strengths and weaknesses, and historically considered comparable in clinical utility[17]. However, recent guidelines increasingly favor automated static threshold perimetry (Humphrey) for evaluating visual fields in pituitary disease, owing to its superior sensitivity in detecting subtle bitemporal defects [15, 34, 35]. The choice of perimetry method was not under our control, and the lack of standardized metrics for direct comparison further complicated the analysis. Consequently, we had to group patients into broad categories rather than perform more detailed analyses. Further, missing data and exclusions significantly reduced the subgroup sizes. A well-documented limitation of visual field testing is its dependence on both patient cooperation and examiner proficiency. To mitigate this, all cases deemed unreliable by the examiner were defined as missing. Additionally, a significant number of patients were excluded due to ocular comorbidities that may affect visual assessment. Although these patients were re-included in the analysis of postoperative improvement, the requirement for reliable pre- and postoperative assessments limited the final sample size. Nonetheless, the use of strict criteria enhances the stringency of the analyzed data.

The duration of visual impairment before surgery is a challenging variable to assess in many studies including ours. Previous reports have identified short duration as a positive prognostic factor for recovery[43]. While improvement often occurs rapidly after surgery, within minutes to a few days, further improvement may continue over subsequent months[10, 24]. It can thus not be excluded that some patients in this study had further improvement after the 3-month follow-up. However, as patients were categorized broadly into improvement, deterioration, or no change, it is arguably unlikely that individuals showing no improvement within the initial 3 months would experience substantial delayed recovery.

The definition of parasellar invasiveness (defined as Knosp III-IV) is not unproblematic since invasiveness cannot be excluded also in Knosp II. However, non-invasive tumor is seldom seen in Knosp III, why separating invasive from non-invasive tumor is still considered to be best achieved by distinguishing between Knosp II and Knosp III [21, 40, 45].

If our results are translated into clinical practice, they confirm the importance of decompressive surgery in large tumors with extrasellar extension. Considering the postoperative visual outcomes in this study, in combination with previous reports, age appears to be a factor negatively influencing visual prognosis. Although ISP was statistically associated with increased risk of postoperative visual acuity impairment, the lack of correlation with other visual parameters, combined with potential overestimation due to small sample size, calls its clinical relevance into question. Thus, the value of routinely performed intraoperative ISP measurements with the aim of predicting postoperative prognosis remains uncertain. Even though female sex did not correspond with worse prognosis after surgery compared with male sex, any differences in preoperative symptoms may be of clinical importance, to ensure qualified and equal treatment decisions.

## Conclusion

ISP does not appear to be a major risk factor for visual or cranial nerve symptoms, nor for postoperative prognosis following decompressive pituitary surgery. While our findings suggest a possible association between elevated ISP and postoperative visual acuity impairment, this observation requires further validation. Tumor size and suprasellar extension are established risk factors for preoperative visual symptoms, while increasing age is associated with poorer visual outcomes. Female sex was linked to higher risk of preoperative visual deficits but showed no association with surgical prognosis. Parts of our results may be used for understanding postoperative results and risk factors; this could be considered in preoperative patient information.

## Data Availability

Original data are held by the authors and are available on request.

## Abbreviations

ISP: intrasellar pressure
CN: cranial nerve
NFPA: non-functioning pituitary adenoma
MRI: magnetic resonance imaging
AMD: age-related macular degeneration
PCO: posterior capsule opacification
uOR: unit odds ratio
OR: odds ratio
